# Introducing a Chair-Side Novel Approach to Reach Evidence-based Periodontal Information in the Daily Periodontal Practice

**DOI:** 10.4021/jocmr2009.09.1262

**Published:** 2009-10-16

**Authors:** Aous Dannan

**Affiliations:** a Department of Periodontology, Faculty of Dental Medicine, Witten/Herdecke University, Witten, Germany. Email: aousdannan@yahoo.com

## Abstract

**Background:**

Evidence-based healthcare is not an easier approach to patient management, but should provide both clinicians and patients with greater confidence and trust in their mutual relationship. The intellectual embrace of evidence-based methods, coupled with clinical expertise and consideration of the patients individual uniqueness and requirements, is needed for all periodontal therapists if optimum care is the goal. One important element of evidence-based decision making in periodontology is the systematic review. Systematic reviews usually provide the periodontist with the highest level of evidence which should be taken into consideration when constructing any treatment plan in the dental clinic. However, reaching systematic reviews might be a time-consuming procedure that needs further personal skills.

**Methods:**

In this paper, a chair-side novel approach to facilitate the incorporation of systematic reviews into daily periodontal practice is presented. It is based on three simple tools, namely, a list of suitable periodontics-related key words, a data bank of all up-to-date published systematic reviews in periodontology, and hand-made paper sheets to match the key words with their related systematic review statements.

**Results and Conclusions:**

A primary validation of this method indicated the simplicity in learning and application.

**Keywords:**

Chair-side; Evidence-based medicine; Periodontology; Systematic review

## Introduction

Evidence-based medicine (EBM) is defined as the “integration of the best research evidence with clinical expertise and patient values” [1]. The use of evidence to help guide clinical decision is not new. However, the methods of generating high-quality evidence (e.g. randomized controlled trials), the integration of systematic reviews and meta-analysis, and the ways for accessing the evidence (e.g. electronic databases) are all new [2,3].

The American Dental Association (ADA) has defined evidence-based dentistry (EBD) “as an integration of systematic assessments of clinically relevant scientific evidence, relating to the patients oral and medical condition and history, with the dentists clinical expertise and the patients treatment needs and preferences” [4]. This definition is now incorporated in the ADA Accreditation Standards for Dental Education Programs.

According to the Medical Subject Headings (MeSH), Periodontics, also known as Periodontology, is a dental specialty concerned with the histology, physiology, and pathology of the tissues that support, attach, and surround the teeth, and of the treatment and prevention of disease affecting these tissues.

The practice of periodontology continues to increase in complexity. Developments in therapies and techniques, changing socio-demographic patterns, increasingly knowledgeable health care consumers, and the explosive information all are placing greater demands on clinical decision making [5]. As health care practitioners, it is important to offer the best possible care for patients.

Evidence-based periodontology (EBP) could be simply defined as “the application of evidence-based health care to periodontology” [6]. It is a tool to support decision making and integrating the best evidence available with clinical practice.

It is supposed that translating evidence-based decision making into clinical action is based on many abilities and skills [1], such as:

1. Converting information needs and problems into clinical questions so that they can be answered.
2. Conducting a computerized search with maximum efficiency for finding the best external evidence with which to answer the question.
3. Critically appraise the evidence for its validity and usefulness.
4. Apply the results of the appraisal in clinical practice.
5. Evaluate the process and ones performance.

One important element of evidence-based decision making in periodontology is the systematic review. Systematic reviews and meta-analysis using two or more randomized controlled trials of human subjects are considered to present the highest level of evidence, or the gold standard in medicine [7,8]. A high quality systematic review can: 1) find and summarize all available studies, 2) provide an objective assessment of the quality or research and in particular the degree of protection from bias within the original studies, 3) estimate research effects across multiple studies with meta-analysis, and 4) overcome limitations of underpowered studies in detecting a true difference if such a true difference really exists.

However, the process of reaching the highest level of evidence, namely a systematic review, in the daily periodontal practice, and then applying it on patients usually takes a long time due to the need for some computer skills to deal with the electronic databases (e.g. PubMed) and then to find out the requested systematic review concerning a specific debated point in periodontology. Such skills are not always available, and, moreover, the existence of a computer which is connected to the internet is not the usual case in many dental clinics.

In this paper, we introduce a manual chair-side, time saving novel approach to enable the dental practitioners in general, and the periodontists in specific, to reach any systematic review in the field of periodontology in few simple steps and without having special skills or using computerized search engines as prerequisites.

## Materials and Methods

### Tools' design

**Figure 1:**
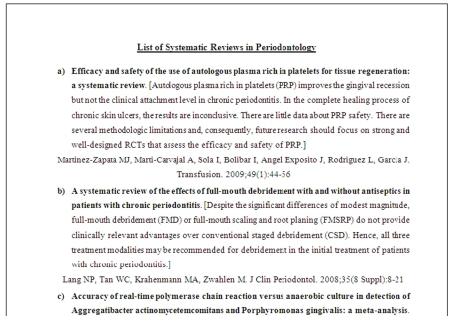
Organization of the list of systematic review statements in periodontology.

Under the website of the ADA-Center for Evidence-based Dentistry (http://ebd.ada.org/Default.aspx), the database of systematic reviews in periodontics, and the related PubMed links were used to collect all its statements up to August 2009. They were picked up, organized by date starting from the latest systematic review, and alphabetically numbered from (a) to (z), and then from (aa) to (zz), and so on until reaching the last systematic review (i.e. nnnnn). A printed copy named "List of Systematic Reviews in Periodontology" was then initiated by means of these statements. Every statement consisted of: 1) the title of systematic review as posted on PubMed, 2) a conclusion, and in some of the cases, extracted parts of the results of the systematic review with the conclusion, and 3) the reference (Author(s), journal name, year, volume, issue, and pages). An example of the statements organization is shown in Figure 1.

**Figure 2:**
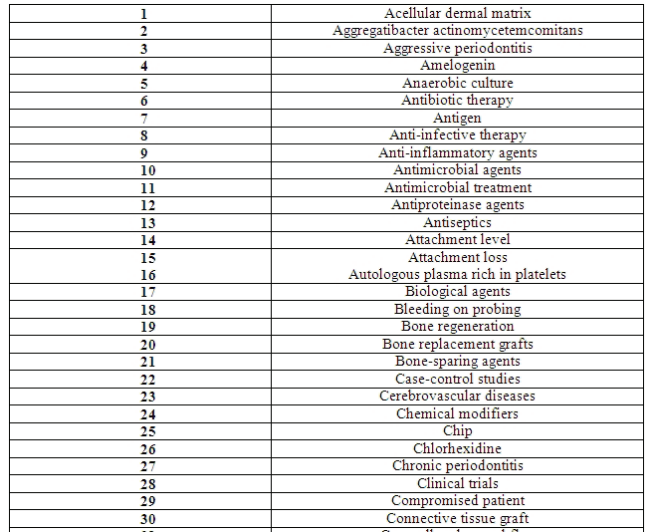
The key words’ list.

At the next step, all important key words were extracted from the titles which exist in the previous list (e.g. autologous plasma rich in platelets, tissue regeneration, full-mouth debridement, antiseptics, chronic periodontitis, etc). All these key words were coordinated alphabetically in a suitable table and numbered (Fig. 2).

Thereafter, every key word from the previous list was searched for in the list of systematic review statements again in order to register how many times a key word was mentioned among the statements. To date, 153 key words could be extracted from 118 systematic reviews.

**Figure 3:**
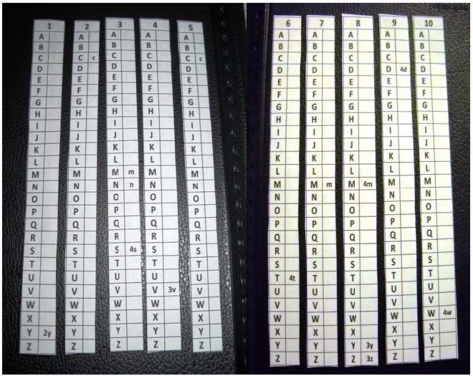
The numerated key words as manual tools on separate sheets.

Every key word, referred to by its own number, was then set in a small table above vertically-constructed alphabets on separate sheets. In every single table, the frequency of a key words appearance among the systematic review statements could be shown next to the related letter (Fig. 3). For instance, under the key word #1, we can see (2y). It means that this key word (i.e. acellular dermal matrix) is mentioned under the statement (yy) in the systematic review list, which is titled “Acellular dermal matrix for mucogingival surgery: a meta-analysis”. By this way, it can also be f ound that the key word #3 is mentioned under the statements (m), (n), and (ssss), and that the key word #8 is mentioned under the statements (mmmm), (yyy), and (zzz) etc…

### Conducting a search for specific systematic review

By means of the previously-described research tools, and in order to find out a systematic review statement which is related to a specific field in periodontology, the following steps should be followed:

1. Make decision on a specific point/idea/concept/question in periodontology to be searched for.
2. Select out suitable related key words which may refer to this point/idea/concept/question.
3. Refer to the related key words’ numbers in the key words list.
4. Pick up the pursuant numbered sheets (described above) and put them side by side .When a shared repeated letter among the sheets is noticed, this means that a systematic review which contains the previously-selected key words does exist. If a shared letter is not noticed, this means that a systematic review which contains all of the previously-selected key words does not exist. In this case, individual systematic reviews could be found which contain the selected key words.
5. Simply refer to the “List of Systematic Reviews in Periodontology” and choose the suitable statement according to the letter which was extracted in the previous step.

To make the whole procedure more understandable, two examples are demonstrated:

#### Example 1

**Figure 4:**
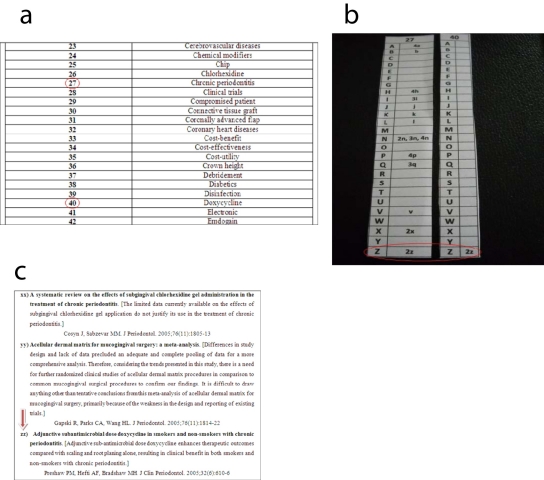
Conducting a search for specific systematic review. (a) choosing suitable key words (i.e. 27 and 40). (b) both key words are mentioned in the statement (zz). (c) referring to statement (zz) in the list of systematic reviews.

Supposedly we would like to find out the latest up-to-date systematic review statement(s) considering the usage of Doxycycline as adjunct procedure for the treatment of chronic periodontitis. In this case, the most likely key words to be appointed are “Doxycycline” and “chronic periodontitis”. In the key words’ list, we refer to the related key words’ numbers. Here, it can be found that “chronic periodontitis” has the number “27”, and that “Doxycycline” has the number “40” (Fig. 4a). We pick up the pursuant numbered sheets (i.e. 27 and 40) and put them side by side (Fig. 4b). It will be noticed that both sheets share the statement “2z” (also recognized as “zz”). We refer to the “List of Systematic Reviews in Periodontology” and choose the statement “zz”. In this case, it will be found that the systematic review statement which contains both key words “Doxycycline” and “chronic periodontitis” is titled “Adjunctive subantimicrobial dose doxycycline in smokers and non-smokers with chronic periodontitis”. In this systematic review, conducted by Preshaw et al. (2005), it is shown that adjunctive sub-antimicrobial dose doxycycline enhances therapeutic outcomes compared with scaling and root planing alone, resulting in clinical benefit in both smokers and non-smokers with chronic periodontitis (Fig. 4c).

#### Example 2

Supposedly we would like to find out the latest up-to-date systematic review statement(s) considering the benefit of using enamel matrix derivative for the treatment of periodontitis. In this case, the most likely key words to be appointed are “enamel matrix derivative” and “periodontitis”. In the key words’ list, we refer to the related key words’ numbers. Here, it can be found that “enamel matrix derivative” has the number “43”, and that “periodontitis” has the number “102”. We pick up the pursuant numbered sheets (i.e. 43 and 102) and put them side by side. It will be noticed that there are three systematic reviews related to “enamel matrix derivative”, which are “ccc”, “rrr”, and “yyyy”. However, it will be noticed that these statements do not contain the key word “periodontitis” at all because there are no shared letter between both sheets. We refer to the “List of Systematic Reviews in Periodontology” and choose the three statements mentioned above.

## Results

To primarily assess the validation of the current method, two periodontists, away from each other, were asked, by means of the current research tools, to find systematic reviews regarding 3 topics in periodontology: 1) the benefit of full-mouth debridement in the treatment of periodontitis, 2) a possible relationship between periodontal disease and stroke, and 3) the use of antiseptics in the treatment of periodontitis. They were firstly instructed how to conduct a search and how to use the tools. Regardless of the way used to find the requested systematic reviews, both periodontists claimed that they could reach the information they looked for. Moreover, they claimed that the information demonstrated were clear, understandable, and could be used as backgrounds when planning a treatment and/or when such information are needed to explain some points to the patient in the clinic.

## Discussion

High quality research and the use of evidence are fundamental to both evidence-based periodontology and traditional periodontology. The differences between these approaches emanate from how research informs clinical practice. Evidence-based periodontology is built upon developments in clinical research design throughout the 18th, 19th and 20th centuries [9,10,11]. It depends on using a more transparent approach to acknowledge both the strengths and the limitations of the evidence. An appreciation of the level of uncertainty or imprecision of the data is essential in order to offer choices to the patient regarding treatment options. Evidence-based periodontology also attempts to gather all available data and to minimize bias in summarizing the data [6]. Clinicians must understand the importance of the research question, study design, and outcomes in order to apply the best available research to patient care [12].

Systematic reviews are considered to be important elements of evidence-based periodontology. The description of systematic reviews as providing the highest level of evidence is widespread [13], and a realistic understanding of what a systematic review can provide is important for the appropriate use of this type of evidence.

Systematic reviews usually provide the periodontist with the highest level of evidence which should be taken into consideration when constructing any treatment plan in the dental clinic. They are also useful to give the patients a comprehensive suitable conclusion regarding any specific topic related to periodontal diseases/treatment/situations (e.g. a possible relationship between bad oral hygiene and preterm birth, or between periodontal disease and heart diseases). Although the description of systematic reviews as providing the highest level of evidence could raise expectations that may or may not be fulfilled [6], we believe that chair-side systematic reviews are important in the daily dental practice, and that conducting any treatment should be achieved under the light of such kind of evidence.

In order to facilitate practicing evidence-based periodontal treatment by means of the previous concepts, we created a printed data bank of all systematic reviews which are related to periodontology up to August 2009, a suitable key words’ list, along with simple manual tools which can be easily used in the dental clinic. We believe that such method may encourage the periodontist to conduct a simple search without complaining of wasting time to start an electronic search on the internet that needs further skills.

The current research approach is so designated, that not only “matched and shared key words” could be found, but even single key words related to single statements. For instance, a search for systematic reviews related to [periodontitis and stress] can be conducted by using either both key words or just one of them. In both ways, the practitioner will find the information he needs, but the ability to find a shared statement which contains both key words would narrow the range of search, and would subsequently save more time.

In the second example (described above), one may choose to look for “Emdogain”, which is the most famous commercial name as “enamel matrix derivative”, instead of Enamel matrix derivative. Here, it must be noted that a limitation of the search is created, and one may only reach fewer systematic reviews than the case when “Enamel matrix derivative” is chosen instead. Another example could be using the key word “periodontal disease” instead of “periodontitis”, and vice versa, in order to reach the requested statement. Such tips must be taken into account, and further trials might be requested to find the final targeted systematic review statement(s).

This research approach is valid not only for one or two key words, but also for unlimited number of them. For instance, one can look for a systematic review by means of the key words “Guided tissue regeneration” and “Periodontal defect”, or by means of the key words “Guided tissue regeneration” and “Periodontal defect” and “Infrabony defects” and “Intrabony defects”. This could enable the practitioner to reach the target more accurately.

According to the validation test of the current search method, it seems to be that the practitioner has to be first familiar with the tools he/she will use before conducting a specific search for systematic reviews, and that every practitioner will be able to develop his/her own manner in conducting a search for systematic reviews by means of the current method.

A very important point related to the current approach is that it is updateable. The “List of Systematic Reviews in Periodontology” can be easily refreshed by means of the ADA-database of systematic reviews in periodontology, which is posted under the website of the ADA-Center for Evidence-based Dentistry (http://ebd.ada.org/Default.aspx). This database is updated quarterly.

### Conclusions

Periodontology has been the leader among dental specialties in embracing the concepts of evidence-based decision making. However, a significant problem in clinical dentistry is the delay between advances in research findings and their incorporation into daily clinical practice.

Current efforts focus on producing summaries of studies, and, appraising and incorporating the quality of the research. These rigorous analyses are called systematic reviews, which are important for practitioner in terms of treatment planning, patient-dentist information exchange, and critical appraisal of new treatment methods.

In order to facilitate the incorporation of systematic reviews into daily periodontal practice, we developed a chair-side novel approach based on three simple tools: 1) a data bank of all up-to-date published systematic reviews in periodontology, 2) a list of suitable periodontics-related key words, and 3) suitable paper sheets to match the key words with their related systematic review statements. A primary validation of this method indicated the simplicity in learning and application.

However, we believe that further investigations are mandatory in order to validate this new method so that it would be further shared out among periodontists. When such approach has been proven to be completely accepted, other specialties might benefit from it and bring it into application.

## References

[1] Straus S, Richardson W, Paul G, Haynes R (2005). Book chapter. Evidencebased medicine: How to practice and teach EBM.

[2] Davidoff F (1999). In the teeth of the evidence: the curious case of evidence-based medicine. Mt Sinai J Med.

[3] Davidoff F, Case K, Fried PW (1995). Evidence-based medicine: why all the fuss?. Ann Intern Med.

[4] American Dental Association (2002). ADA policy on evidencebased dentistry. professional issues and research, ADA guidelines, positions and statements. http://www.ada.org.

[5] Worthington H, Needleman I (2005). Evidence-based periodontal disease prevention and treatment: introduction. Periodontol 2000.

[6] Needleman I, Moles DR, Worthington H (2005). Evidence-based periodontology, systematic reviews and research quality. Periodontol 2000.

[7] Forrest J, Miller S, Newman M, Carranza F (2006). Introduction to evidence-based decision making. Carranza’s Clinical Periodontology. 10th ed.

[8] Manchikanti L, Benyamin RM, Helm S, Hirsch JA (2009). Evidence-based medicine, systematic reviews, and guidelines in interventional pain management: part 3: systematic reviews and meta-analyses of randomized trials. Pain Physician.

[9] Needleman I, Clarkson J, Harrison J, Ismail A, Needleman I, Worthington H (2003). Introduction to evidence based dentistry. Evidence Based Dentistry for Effective Practice.

[10] Rangachari PK (1997). Evidence-based medicine: old French wine with a new Canadian label?. J R Soc Med.

[11] Swales J (2000). The troublesome search for evidence: three cultures in need of integration. J R Soc Med.

[12] Fisher CG, Wood KB (2007). Introduction to and techniques of evidence-based medicine. Spine (Phila Pa 1976).

[13] La Caze (2009). Evidence-based medicine must be. J Med Philos.

